# Central Bradypnea and Ataxic Breathing in Myotonic Dystrophy Type 1 – A Clinical Case Report

**DOI:** 10.3233/JND-221652

**Published:** 2023-05-02

**Authors:** Oliver Summ, Christian Mathys, Teresa Grimm, Martin Groß

**Affiliations:** a Department of Neurological Intensive Care and Rehabilitation, Evangelisches Krankenhaus Oldenburg, Oldenburg, Germany; b Faculty of Medicine and Health Sciences, Carl von Ossietzky University Oldenburg, Oldenburg, Germany; c Department of Radiology and Neuroradiology, Evangelisches Krankenhaus Oldenburg, Oldenburg, Germany; d Research Center Neurosensory Science, Carl von Ossietzky University Oldenburg, Oldenburg, Germany; e Department of Diagnostic and Interventional Radiology, University of Düsseldorf, Germany; f Research Network on Emergency and Intensive Care Medicine Oldenburg, Faculty of Medicine and Health Sciences, Carlvon Ossietzky University Oldenburg, Oldenburg, Germany

**Keywords:** Myotonic Dystrophy Type 1, breathing disorder, breathing regulation, clinical case report

## Abstract

**Background:**

The occurrence of obstructive and central sleep apnea syndromes, ventilator pump failure and reduced hypercapnic ventilatory drive in myotonic dystrophy type 1 (DM1) is well established, and there are indications for an impairment of the hypoxic ventilator drive, too. Yet, it is still unknown, to which extent the respiratory rhythm is affected by DM1, thus if a central bradypnea, cluster breathing or ataxic (“Biot's”) breathing can occur. Additionally, the causes of the impairment of the central respiratory drive in DM1 are not known.

**Case Presentation:**

We present the case of a tracheotomized female patient with DM1 with central bradypnea and ataxic breathing. A 57-year-old woman with DM1 was admitted to our Neurointensive Care Unit (NICU) due to refractory tracheobronchial retention of secretions resulting from aspiration of saliva. Due to a combination of chronic hypercapnic respiratory failure, severe central bradypnea with a minimal breathing frequency of 3 per minute and ataxic breathing a pressure-controlled home ventilation was initiated.

**Conclusions:**

In our patient central bradypnea and ataxic breathing possibly were respiratory sequale of DM1, that may have been caused by pontine white matter lesions affecting the pontine respiratory nuclei. From a clinical viewpoint, polygraphy is a suitable tool to objectify disorders of the respiratory rhythm in DM1 even in tracheotomized patients. Clinical studies combining respiratory diagnostics as polygraphy, transcutaneous capnometry and blood gas analysis with brain magnetic resonance imaging (MRI) are required to better understand disorders of respiratory regulation in DM1, and to identify their anatomical correlates.

## ABBREVIATIONS

CTG=: cytosine-thymine-guanine
DM1=: myotonic dystrophy type 1
FEES=: fiberoptic endoscopic evaluation ofswallowing
MI-E =: mechanical insufflator-exsufflator
MRI=: magnetic resonance imaging
NICU=: neurological intensive care
PEG=: percutaneous endoscopic gastrostomy

## INTRODUCTION

Myotonic dystrophy type 1 (DM1) is an autosomal-dominant genetic disorder due to an expansion of a cytosine-thymine-guanine (CTG) repeat in the dystrophia myotonica protein kinase gene of chromosome 19q13.3 [1]. DM1 can cause extramuscular symptoms such as diabetes cataract and hypogonadism [2–4]. Neuropathological studies have revealed cerebral ,,1) protein and nucleotide deposits; 2) changes in neurons and glia cells; and 3) white matter alterations“ [5, Fig. 5]. In recent years, DM1 has thus been acknowledged as a brain disease [6].

The clinical phenotype is variable, generally associated with the length of the number of CTG-repeats and progresses from generation to generation [4]. Cases with severe neuromuscular respiratory failure and need for invasive ventilation can occur as well as much less severe phenotypes. A landmark review [7] showed, that there is a wide spectrum of respiratory impairments in DM1: obstructive and central sleep apnea syndromes, neuromuscular respiratory failure and impairment of the hypercapnic ventilatory response. A study by Carrol, Zwillich and Weil [8] demonstrated an impairment of the hypoxic respiratory drive. In a study with 69 participants, Poussel et al. [9] found out that the breathing response is reduced in reaction to CO2 in DM1 and that this does not correlate with the lung function. They concluded that this dysfunction of breathing regulation has a central cause. Yet, it is unknown, if central bradypnea, cluster breathing or ataxic(,,Biot's“) breathing can occur. Additionally, the causes of the impairment of the central respiratory drive in DM1 are unknown.

## METHODS

We present a case of a tracheotomized female patient with genetically confirmed DM1 with central bradypnea as well as alveolar hypoventilation. Furthermore, we analyze and discuss polygraphic data and imaging data with regard to respiratory regulation. This clinical case report is conducted in accordance with the case report guidelines that were established by Gagnier et al. [10].

## CASE PRESENTATION

## Participant information

A 57-year-old obese (BMI 39,1) and tetraparetic woman was referred from a regional hospital due to insufficient cough and refractory tracheobronchial retention of secretions. She was under treatment in our Neurointensive Care Unit (NICU) between May 2020 and September 2020, where we confirmed the genetic diagnosis of DM 1 with a CTG-repeat-expansion of > 500 to 800 copies. Before admittance she lived in a nursing home and was using a wheeled walking frame and a wheelchair. Her father also lived with DM1. Her two children had died within 24 hours after birth. The participant provided an informed consent to publish health data including polygraphic and imaging data.

## Diagnostic assessments

The diagnostic standard in patients admitted to our NICU with insufficient cough, tracheobronchial retention of secretions and imminent respiratory failure comprises blood gas analysis and fiberoptic endoscopic evaluation of swallowing (FEES). The x-ray of the chest revealed a reduced depth of inspiration but did not reveal any evidence for an emminent pneumonia, emphysema, atelectasis or restrictive components. In the previous clinic a cerebral magnetic resonance imaging (MRI) (including thin-slice isotropic T1- and FLAIR-weighted images; optional gadolinium-enhanced T1-weighted images as needed) was performed to identify brain stem lesions. This was conducted because an acquired central hypoventilation syndrome was suspected due to hypercapnic respiratory failure without tachypnea or dyspnea. The imaging of the central nervous system was supplemented by MRI of the upper cervical spine. Polygraphy was used to analyze the respiratory rhythm as apnea or desaturations are present.

The electroencephalography revealed a background activity of 8 Hz without findings of epilepsy related potentials and no focal findings but an underlying theta activity that was present for more than 50% of the revulsion and was attributed to the mild hypercapnic state of the patient. After initiation of mechanical ventilation the vigilance was elevated, due to lack of consequences the EEG was not repeated.

Electromyographic and electroneurographic investigation were in consistency with the diagnosis of a DM1.

## Clinical findings

In the neurological examination the patient presented with anisocoria (left > right), but regular reaction of the pupil, and dysarthria. There was no sign of meningism, there were no additional pathologies seen in the investigation of the cranial nerves. A motor weakness was seen pronounced in the upper vs. the lower limb (grade 3 vs grade 4). Muscle reflexes presented symmetrical, weak. She demonstrated with a poor performance in all motor tasks (fingerpointing, evaluation for diadochokinesis etc.). There was no sign of sensory deficit. She was able to manage short distances by usage of a 4-wheeled walker, otherwise dependent from a wheel-chair.

Hypercapnic ventilatory failure with respiratory acidosis and metabolic compensation was present on admission: The blood gas analysis on admission showed a pH of 7,36, a pCO2 of 50 mmHg, a pO2 of 63 mmhg and a base excess of 1,7 mmol/l. The Saturation was measured at 98% as a first measurement on admission under nasal supplementation of 2 liters of oxygen per minute. One day later we found a pH of 7,40, a pCO2 of 56 mmHg, a pO2 of 68 mmhg and a base excess of 7,7 mmol/l.

The patient was pretreated in another hospital with trimethoprim/sulfamethoxazole (1:5) and did not show signs of acute infection on admission to our hospital. The laboratory testing did reveal a slightly elevated leucocyte count (11.5 /nl) with a procalcitonin of 0.12 ng/ml and CRP of 11.7 mg/dl. Antibiotic treatment was changed to piperacilline /tazobactame for consecutive 8 days with and adequate normalization of laboratory markers indicative for infection. The microbiological screening did not help to identify any bacteria from catheters or secretions at this stage, however after tracheostomy pseudomonas aeruginosa was identified from the endotracheal secretion.

**Fig. 1:**
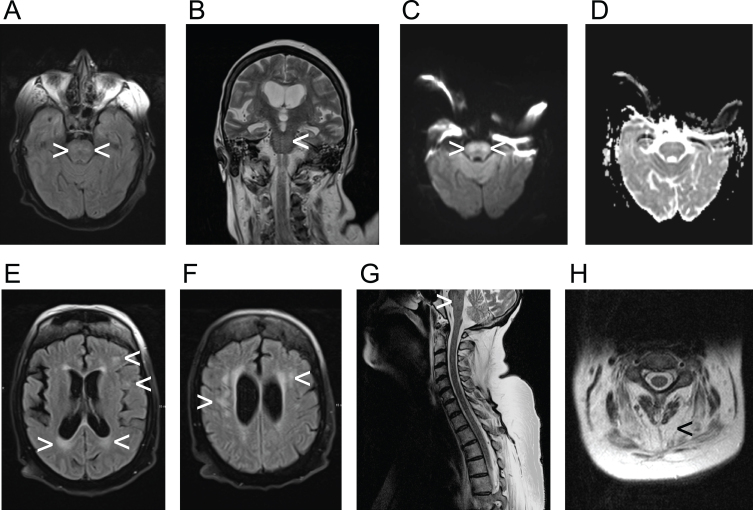
Demonstration of radiological findings in the patient, diagnosed with myotonic dystrophy type 1, utilizing various acquisition techniques. A) fluid-attenuated inversion recovery (FLAIR), axial view; B) coronal T2-weigthed image; C) axial view, diffusion-weighted magnetic resonance imaging (DWI); D) axial view, apparent diffusion coeffizient (ADC); E) and F) fluid-attenuated inversion recovery (FLAIR), axial views; G) T2-weigthed image, saggital view; H) T2-weigthed image, axial view with highlighted sign of muscular atrophy. Arrowheads are implemented to highlight pathologies exemplary. The participant provided an informed consent to publish health data including imaging data.

**Fig. 2:**
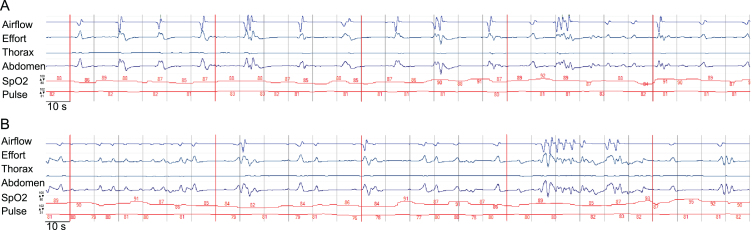
Display of an episode of the polygraphic recording showcasing central bradypnea. The time interval from one to another vertical line is 10s, the interval between two neighboring vertical red lines marks a 60s timeframe. The default range of the measurement of SpO2 and heart rate of the pictured measurement is given with respect to the upper and lower limit. SpO2 is displayed as a percentage, pulse as numbers of heart rate in beats per minute. The categories airflow, effort, thorax(-movement), abdomen(-movement) and activity are displayed in arbitrary units. Section a) demonstrates bradypnea and prolonged expiration, section b) demonstrates irregular variability of breathing effort and timing, consistent with ataxic (“Biot's”) breathing. The participant provided an informed consent to publish health data including polygraphic data.

Severe dysphagia with aspiration of saliva was identified by FEES. An MRI from the referring hospital already had shown subcortical and pontine signal alterations (Fig. 1). Polygraphy showed a bradypneic episode, lasting 2 hours and 36 minutes with a minimum breathing frequency of 3/minute (Fig. 2) and episodes of ataxic breathing with irregular variability of breathing effort and timing. The in parallel performed trancutaneous canpnography over a period of 10,3 hours revealed a minimum pCO2 of 45 mmHg, median of 52 mmHg, maximum of 61 mmHg with 9 events of a pCO2 > 50 mmHg of a duration of more than 5 minutes.

We found no competing factors leading to bradypnea, the thyroid and the regulating hormones were checked normal, there was an euglycaemia, no opioids were given in timely context of the polygraphy, no other drugs with potential impact on the breathing pattern were applied. Drugs applied prior to admittance to our hospital comprised trimethoprim/sulfamethoxazole (1:5) due to a pre-diagnosed pneumonia, apixaban due to the history of a pulmonary embolism, foliac acid due to a deficit, dorzolamide/timolol due to glaucoma, she received no sedatives. In order to exclude autoimmune or paraneoplasic involvement we performed serologic screening which came back negative for all screened antibodies, the rheumatological workup and additional laboratory testings was also negative (e.g. copper in serum, serum electrophoresis). A lumbar puncture was not performed as the clinical findings did correspond to the patients’findings.

## Interventions

The patient was electively tracheotomized one day after admission to our NICU due to salivary aspiration, tracheobronchial retention of secretions and threatening aspiration pneumonia. In addition, the patient was –according to the German guidelines for home ventilation [11] concerning hypercapnic respiratory failure in neuromuscular disease –put on invasive ventilation at night and during daytime sleep. Regular use of a mechanical insufflator-exsufflator (MI-E) and a mesh nebulizer using sodium chloride 0,9% was installed to foster elimination of secretions. Nutrition was provided by a percutaneous endoscopic gastrostomy (PEG). The patient received botulinum toxin injection into the salivary glands to treat chronic sialorrhea with salivary aspiration.

During the stay in the NICU, the patient received neurological-neurosurgical early rehabilitation by an interdisciplinary team of doctors, nurses, respiratory therapists, speech- and language therapists, occupational therapists and other disciplines. One of the therapeutic strategies was to mobilize the patient in a positioning wheelchair.

## Outcomes

The invasive ventilation resulted in a stable clinical situation with adequate communication and without daytime sleepiness. At the beginning, the patient was mobilizable for a maximum of 30 minutes per day. At discharge, she was independently mobile with an assistant on a high walker and with her power wheelchair. Moreover, the patient was still fed by PEG, but she was able to eat once a day under supervision. She had also been provided with assisted communication but preferred to speak with a speaking valve.

## DISCUSSION

DM1 is a highly complex disorder, not only affecting the respiratory muscles, but also the regulation of breathing. As stated above the occurrence of neuromuscular respiratory failure, obstructive and central sleep apnea syndromes and an impairment of the hypercapnic respiratory drive in DM1 patients is already well established. Only one study has examined the hypoxic and the hypercapnic ventilator response in seven patients with DM1 and found the hypoxic ventilator response more consistently reduced than the hypercapnic ventilator response [8].

Our case with severe central bradypnea and ataxic breathing in DM1 is to the best of our knowledge the first such case to be found in the scientific literature. Polygraphy was used as it comprises measurements not included in a standard NICU monitoring and is able to generate an output of data validated for the diagnosis of breathing irregularities. Despite the extremely low breathing frequency observed during monitoring and polygraphy hypercapnic coma did not result. Preserved residual breathing rhythmicity, intermittent higher breathing frequencies and possibly elevated tidal volumes might have contributed to sustain decarboxylation up to a degree sufficient for survival (Fig. 2).

Nonetheless, our case indicates that impairment of respiratory regulation in DM1 comprises not only reduced ventilatory responses to O2 and CO2 and central apnea, but also a wider spectrum of disorders of the respiratory rhythm. The causes of the impairment of central respiratory regulation in DM1 are as far as we know still unknown. The occurrence of white matter lesions at various cerebral locations has been well established in DM1 [2,3]. While voxel-based morphometry revealed extensive white matter involvement in DM1 including the brain stem in a study by Minnerop et al. [2], infratentorial lesions on FLAIR scans had not been reported in another study by Leddy et al. [3]. Though Leddy et al. mainly studied patients with up to 500 CTG-repeats, while our patient featured with > 500 to 800 CTG-repeats. The MRI of our patient revealed pontine signal alterations in FLAIR, T2 and DWI weighted images, while the ADC weighted images showed no hypointense signal in the pontine region (Fig. 1). The elevated signal in DWI weighted images is thus a T2 shine-through and might be explained by demyelination. There is no sign of an acute infarction. This, again, fits well with the neuropathology of DM1, of which demyelination is a key feature [5]. In line with previous observations the supratentorial white matter lesions were found in a distribution similar one would expect in multiple sclerosis [3]. Lacunes or microbleeds, which are characteristic for small vessel disease, were not observed in our patient. Nevertheless, the pontine signal alterations in our patient, demonstrating as FLAIR-hyperintense white matter lesions, might also have another cause than DM1, e.g. cerebral microangiopathy or osmotic demyelination.

Neuroanatomic and physiological studies have over the last decades well established the role of medullary and pontine regions in the control of breathing mainly in animal models, but also in humans. The Nucleus Kölliker-Fuse and the parabrachial-Komplex (KF-PB) in the dorsolataeral pons are the main pontine regions of interest, and they interact with the medullary centers of respiratory rhythmogenesis [12]. There is growing evidence, that the Nucleus Kölliker-Fuse is not only involved in respiratory phase transitions, e.g. from the inspiratory to the post-inspiratory, and from the postinspiratory to the late-expiratory phase, but also controls the variability of the respiratory rhythm [13–15]. The parabrachial complex plays a key role in the control of the breathing frequency by changes of the duration of expiration [16]. In our patient, breathing frequency, duration of expiration and the variability of the respiratory rhythm are severely impaired (Fig. 2), a constellation possibly caused by lesions in both pontine key regions of respiratory control. Considering the current model of a rhythmogenic network of several pontine and medullary nuclei [17], a dysfunction of the rhythmogenic network in the brain stem has to be assumed in our patient. From a neuroanatomical point of view, it can thus be hypothesized that the pontine alterations of the MRI-signal in our patient represent white matter lesions which cause the polygraphically recorded impairment of the respiratory rhythm.

Therefore, regarding our patient a direct link with DM1 causing pontine white matter lesions, which again causes impairment of the respiratory rhythm can be hypothesized. Alternatively, the pontine MRI-signal alteration is not caused by DM1. However, the impairment of the respiratory rhythm in our patient is either caused by DM1 directly or by her pontine lesions.

## Limitations

A follow-up screening was not conducted. In addition, our case report does not include a statement by the patient about her experience and perspective. This perspective is important as additional needs can be identified and addressed. An interview study showed that patients with DM1 were satisfied with the support concerning their physical symptoms but they wished to receive more psychological an emotional support for them and their partners [4]. We were not able to gain data from pulmonary function due to weakness of bulbar muscles and later also tracheostomy.

## CONCLUSIONS

DM1 can cause ventilator pump failure, obstructive and central sleep apnea, acquired central hypoventilation and possibly central bradypnea and ataxic breathing. From a clinical viewpoint, polygraphy is a suitable tool to objectify disorders of the respiratory rhythm in DM1 even in tracheotomized patients. Mechanical ventilation during sleep may be the best therapeutic option to reverse daytime sleepiness. DM1 might also result in unexpected central neurological deficits caused by white matter lesions.

Clinical studies combining respiratory diagnostics as polygraphy, transcutaneous capnometry and blood gas analysis with brain MRI are required to better understand the complexity of disorders of the respiratory regulation and to identify their anatomical correlates in DM1.

## ETHICS STATEMENT

We hereby confirm that the present study conforms to the ethical standards and guidelines of the journal.

## AUTHOR AGREEMENT

All authors have participated in the article preparation and agreed the contents of the submission.

## CONFLICT OF INTEREST

The authors have no conflict of interest to report.
